# Integrating GWAS and proteome data to identify novel drug targets for MU

**DOI:** 10.1038/s41598-023-37177-y

**Published:** 2023-06-27

**Authors:** Yadong Wu, Jukun Song, Manyi Liu, Hong Ma, Junmei Zhang

**Affiliations:** 1 Department of Oral and Maxillofacial Surgery, The Affiliated Stomatological Hospital of Guizhou Medical University, Guiyang, China; 2 Department of Oral and Maxillofacial Surgery, The Affiliated Stomatological Hospital of Zunyi Medical University, Zunyi, China; 3 Department of Orthodontics, The Affiliated Stomatological Hospital of Guizhou Medical University, Guiyang, 550002 China

**Keywords:** Biomarkers, Drug screening, Biomarkers, Health care

## Abstract

Mouth ulcers have been associated with numerous loci in genome wide association studies (GWAS). Nonetheless, it remains unclear what mechanisms are involved in the pathogenesis of mouth ulcers at these loci, as well as what the most effective ulcer drugs are. Thus, we aimed to screen hub genes responsible for mouth ulcer pathogenesis. We conducted an imputed/in-silico proteome-wide association study to discover candidate genes that impact the development of mouth ulcers and affect the expression and concentration of associated proteins in the bloodstream. The integrative analysis revealed that 35 genes play a significant role in the development of mouth ulcers, both in terms of their protein and transcriptional levels. Following this analysis, the researchers identified 6 key genes, namely BTN3A3, IL12B, BPI, FAM213A, PLXNB2, and IL22RA2, which were related to the onset of mouth ulcers. By combining with multidimensional data, six genes were found to correlate with mouth ulcer pathogenesis, which can be useful for further biological and therapeutic research.

## Introduction

Mouth ulcer (known as oral ulceration) refers to a lesion that forms on the mucous membrane of the oral cavity. It occurs due to the damage caused to both the epithelium and lamina propria. Mouth ulcer is a non-specific term capturing different entities, but the most common one is aphthous ulceration. Children and adolescents are more likely to develop mouth ulcers, which are the most common ulcerative disease in humans^1,2^. Even though mouth ulcers do not pose a significant health burden, they can negatively affect your quality of life and overall health^3–5^. Several factors may contribute to the etiology, such as stress, food, trauma, hormonal imbalances, immune disorders, gastrointestinal disorders, and smoking^6^. The high incidence of mouth ulcers and their adverse effects on quality of life have stimulated much research on the etiology and effective treatment of this disease. Even though several risk factors are simultaneously related to mouth ulcers, the genes responsible for causing mouth ulcers are still unknown. It is therefore necessary to identify molecular features of mouth ulcer pathogenesis to offer a rationale for treatment.

Genome-wide association studies (GWAS) have identified several loci associated with mouth ulcers using high-throughput sequencing technologies^7^. Even with some efforts, the underlying mechanisms attributed to MU risk have remained elusive, making it difficult to translate identified risk loci into clinical interventions. Meanwhile, proteins are the most efficient biomarkers and therapeutic targets^8,9^ Since they are primary components of cellular and biological processes and products of gene expression^10^. Therefore, it is vital to screen the risk proteins in mouth ulcers.

Recently, large-scale quantitative trait loci (QTL) data have been used to examine the association between genotypes and protein abundance (pQTL) and gene expression (eQTL)^11,12^, which has promoted the emergence of statistical methods that facilitate the combination of multidimensional data^13^. Wingo et al.^14^ constructed a new framework named proteome-wide association study (PWAS) to integrate gene and protein expression data with GWAS findings (integrated gene expression data and GWAS results) in depression pathogenesis. In addition, Mendelian randomization (MR) and Bayesian colocalization analyses have been widely employed to screen hub genes by combining QTL and disease-related GWAS data^15,16^. Altogether, By combining GWAS data with multidimensional QTL data, potential genes contributing to mouth ulcers will be identified.

The data from high-throughput proteomics and blood proteomes were combined in this study to identify potential genes related to mouth ulcers. To identify potential protein biomarkers, we used a three-step approach to systematically link protein biomarkers to mouth ulcers. First, we performed PWAS analysis using GWAS data from oral ulcers and a protein quantitative trait locus (pQTL) dataset obtained from blood. Second, the Mendelian randomization (MR) analysis was performed to validate PWAS significant genes. Third, the COLOC method was used to combine the GWAS data and blood pQTL via Bayesian co-localization analysis to determine whether the two correlation signals correlate with a common variation.

## Results

### PWAS identified 35 genes correlated with MU

**Figure 1:**
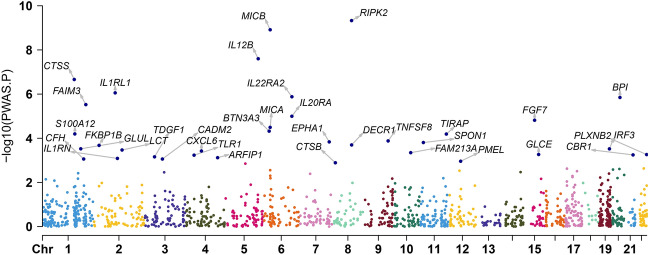
Manhattan plot for the discovery mouth ulcers PWAS integrating the mouth ulcer GWAS with human blood proteomes. Each point represents a single association test between a gene and mouth ulcers ordered by genomic position on the x axis and the association strength on the y axis as the − log10(P) of a z-score test.

**Figure 2:**
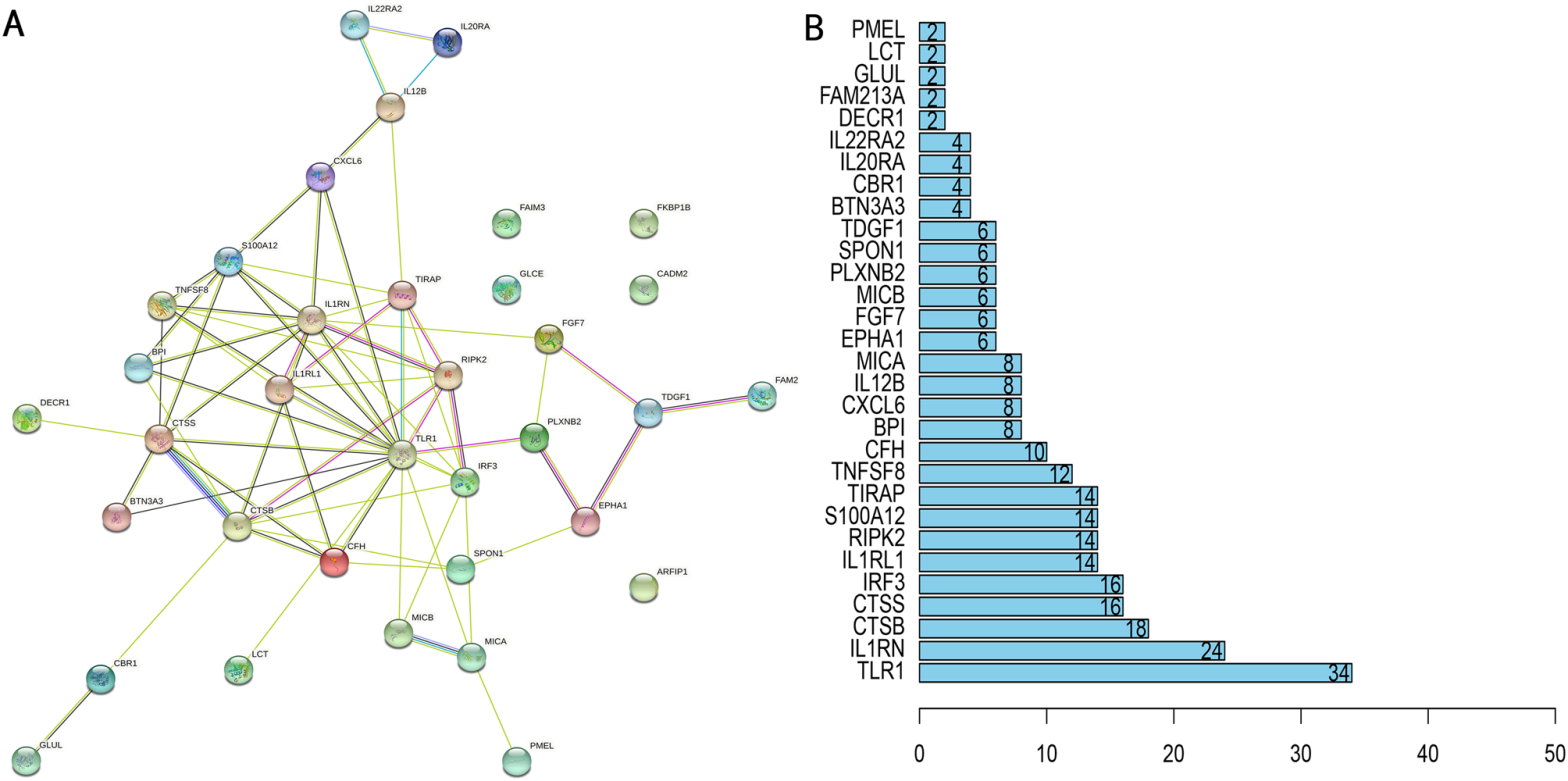
Protein–protein interaction (PPI) among 35 genes screened by the PWAS. (**A**) PPI network; (**B**) hub genes identified by the degree of PPI network.

**Figure 3:**
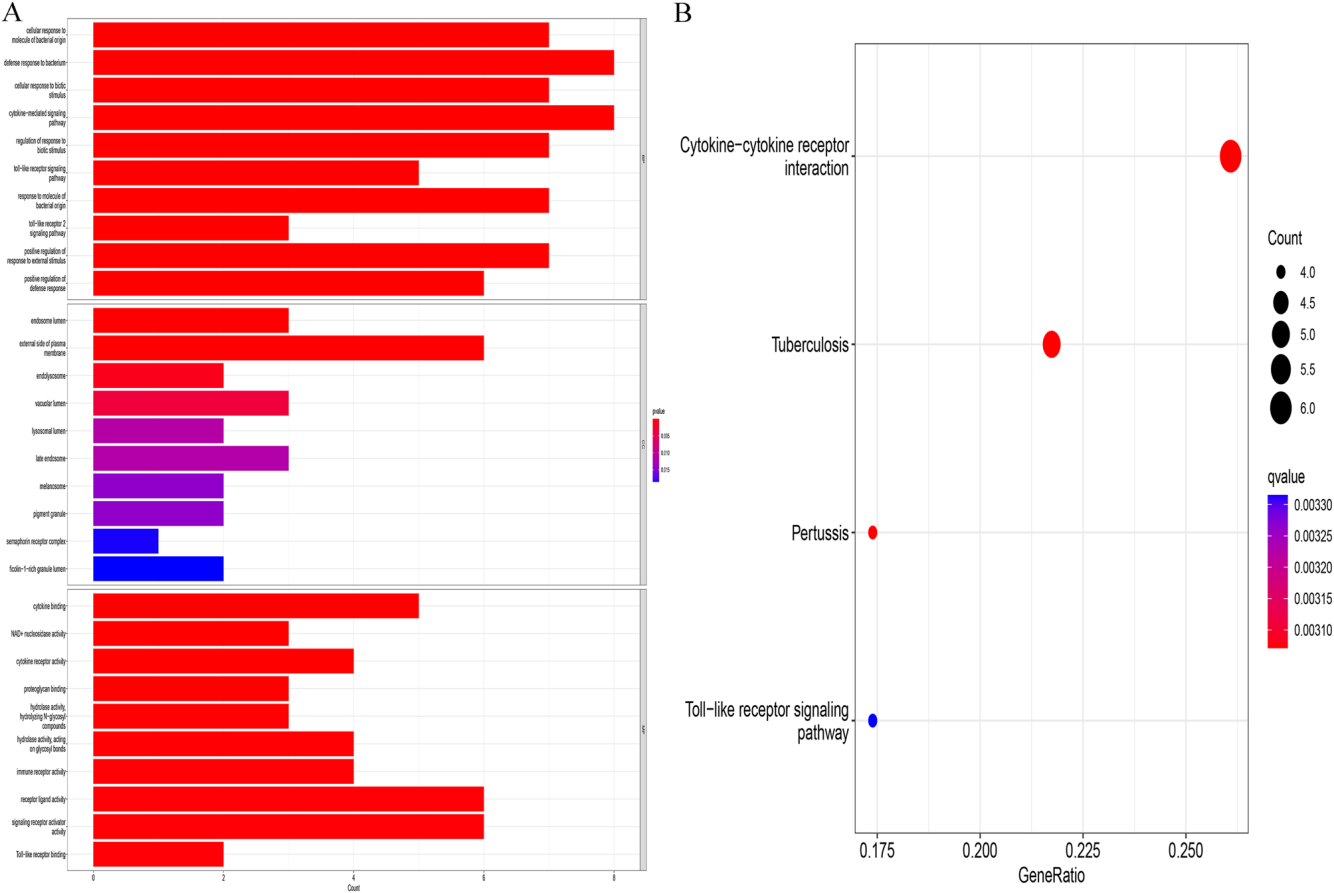
The functional enrichment analysis among candidate genes. (**A**) GO terms; (**B**) KEGG categories.

By combining MU GWAS data with blood proteomes, the FUSION pipeline was used to conduct the PWAS of mouth ulcers^17^. Based on the Bonferroni correction threshold of P 0.05/number of genes analyzed, PWAS identified 35 genes with protein abundances related to oral ulcers (Fig. 1 and Supplementary Table 1). These genetic instruments all had F statistics exceeding 10, which indicates strong instruments^18^. During the study, the researchers created a PPI network (depicted in Fig. 2A and B) and discovered that TLR1, IL1RN, and CTSB were the central genes in the protein interaction network. The GO enrichment analysis demonstrates that 1,170 terms, including 1065 BP terms, 70 CC terms, and 34 MF terms, were enriched. Among these GO categories, defense response to the bacterium, cytokine-mediated signaling pathway, and toll-like receptor signaling pathway was identified (Fig. 3A). The KEGG enrichment analysis^19^ revealed that four KEGG terms were found, including the Toll-like receptor signaling pathway, Cytokine-cytokine receptor interaction, Tuberculosis, and Pertussis (Fig. 3B). Through the above finding, we found that these genes were involved in inflammation.

### MR validates 30 genes correlated with MU using blood pQTL

The majority of proteins analyzed could be instrumented with one or more SNPs. Thus, the Wald ratio method, IVW, and MR egger method were primarily used to estimate MR. MR analysis of blood pQTL and mouth ulcer GWAS identified 30 protein biomarkers, which exhibited strong evidence of association [P < 0.001 (0.05/35)] (Table 1). The proteins encoded by eight genes (PMEL, ARFIP1, FAM213A, GLUL, CADM2, FAIM3, RIPK2, and DECR1) have only one SNP instrumental variable and have a association with mouth ulcers using the Wald ratio method. Five genes have two SNP instrumental variables. Among 23 genes with more than 3 instrumental variables, 11 genes (RIPK2, CXCL6, ARFIP1, TDGF1, TIRAP, TNFSF8, TLR1, GLCE, MICA, and CTSS) have a association with mouth ulcers using the MR egger method. Most of the 11 genes except for CADM2, CXCL6, and ARFIP1 have no horizontal pleiotropy using the inception of MR egger with a P value > 0.05.

**Table 1:** Candidate genes identified by Mendelian randomization.

FILE	IVW								MRE				INT
	ID	n_SNP	F	PVE	OR	LCI	UCI	pval	OR	LCI	UCI	pval	pval
SeqId_8970_9	RIPK2	1	38.068403	0.005251489	1.5015	1.3213	1.7064	4.68E-10	NA	NA	NA	NA	NA
SeqId_17692_2	BTN3A3	7	513.1016058	0.332667158	1.0384	1.0221	1.0549	2.90E-06	1.0907	1.032	1.1527	0.002104989	0.044467728
SeqId_6574_11	FAIM3	1	55.45333656	0.007631417	0.7685	0.6882	0.8582	2.96E-06	1.0625	0.9894	1.141	0.095438554	0.437053838
SeqId_13733_5	IL12B	5	318.7645594	0.181099294	1.0884	1.0504	1.1277	3.01E-06	1.0381	0.9871	1.0917	0.145855911	0.682178135
SeqId_4126_22	BPI	8	268.6442477	0.229778531	0.9557	0.9369	0.9749	8.11E-06	1.0063	0.9611	1.0536	0.788209791	0.021765487
SeqId_16324_38	TLR1	3	115.008651	0.045674446	1.1107	1.06	1.1638	1.05E-05	0.7293	0.3317	1.6035	0.432278637	0.750608964
SeqId_3421_54	TNFSF8	2	124.8032619	0.033461084	1.1191	1.0619	1.1794	2.62E-05	0.9434	0.912	0.976	0.000773348	0.356471325
SeqId_5852_6	S100A12	2	153.0185087	0.04071787	0.9075	0.8663	0.9507	4.25E-05	NA	NA	NA	NA	NA
SeqId_5085_18	IL20RA	2	37.45119254	0.010281865	0.8236	0.7505	0.9038	4.28E-05	NA	NA	NA	NA	NA
SeqId_8007_19	CTSB	6	401.0467364	0.25033398	1.0395	1.0198	1.0597	7.40E-05	NA	NA	NA	NA	NA
SeqId_4487_1	FGF7	2	105.0609899	0.028317861	0.8973	0.8479	0.9495	0.000173362	0.9482	0.8633	1.0415	0.266596183	0.46786433
SeqId_16907_3	CADM2	1	63.46671544	0.008724587	0.828	0.7483	0.9161	0.000253422	1.042	1.0104	1.0746	0.008809747	0.787138542
SeqId_19238_12	GLUL	1	42.60238406	0.005873273	1.2573	1.1106	1.4234	0.000298092	NA	NA	NA	NA	NA
SeqId_3495_15	CXCL6	6	309.1617985	0.204720977	0.9625	0.9424	0.983	0.000381598	0.934	0.7376	1.1827	0.570870614	0.609836798
SeqId_13423_94	FAM213A	1	31.0599254	0.004288825	1.2961	1.1212	1.4983	0.000453355	NA	NA	NA	NA	NA
SeqId_3431_54	EPHA1	6	528.8139051	0.305705662	0.9682	0.9507	0.9861	0.000526486	0.9722	0.9506	0.9942	0.013508973	0.543032243
SeqId_13488_3	ARFIP1	1	680.0861724	0.086184102	1.057	1.0235	1.0917	0.000756065	0.9917	0.9528	1.0322	0.683866271	0.145699908
SeqId_11568_2	FKBP1B	4	362.3539279	0.16741898	1.0431	1.017	1.0699	0.001110212	NA	NA	NA	NA	NA
SeqId_6472_40	PMEL	1	217.6866556	0.029303518	0.9144	0.8664	0.965	0.001135163	1.0287	0.9827	1.0769	0.225624154	0.457867637
SeqId_4159_130	CFH	3	292.072019	0.108372621	1.048	1.0187	1.0781	0.001190992	NA	NA	NA	NA	NA
SeqId_3181_50	CTSS	6	336.4989532	0.218861257	1.0467	1.0174	1.0769	0.001644309	1.0363	0.9546	1.1251	0.394959419	0.844380389
SeqId_9216_100	PLXNB2	9	272.0506841	0.253687746	1.0385	1.0135	1.0642	0.002370096	0.9634	0.7639	1.2151	0.753101904	0.364736195
SeqId_4234_8	IL1RL1	5	495.2230446	0.255714733	1.0532	1.0167	1.0909	0.003978463	0.9532	0.923	0.9843	0.003479931	0.43361207
SeqId_7808_5	GLCE	6	407.0968057	0.253154415	0.9697	0.9488	0.9909	0.005429374	NA	NA	NA	NA	NA
SeqId_4297_62	SPON1	5	264.8158551	0.155206537	0.9686	0.9453	0.9924	0.009926237	0.9774	0.9444	1.0114	0.189965044	0.538546555
SeqId_17151_84	IRF3	6	195.0340163	0.139705777	1.0438	1.0074	1.0815	0.017853371	1.047	1.0047	1.0912	0.02924445	0.62189577
SeqId_12687_2	DECR1	2	73.17078212	0.019893253	0.9224	0.8617	0.9875	0.02030737	0.926	0.8576	0.9999	0.049767006	0.195479532
SeqId_5810_25	TDGF1	10	316.1684401	0.305073397	1.0514	1.0056	1.0993	0.027314813	1.0295	0.9167	1.1562	0.623303462	0.161243876
SeqId_12381_26	CBR1	3	174.0494184	0.067538254	0.9611	0.9273	0.9961	0.029690444	1.1201	1.0483	1.1967	0.000784899	0.024025727
SeqId_5353_89	IL1RN	5	135.2443792	0.085779889	1.0357	1.0023	1.0701	0.035905754	NA	NA	NA	NA	NA
SeqId_9017_58	LCT	3	54.15676474	0.022040419	1.068	1.0011	1.1393	0.046308336	1.0812	0.98	1.1928	0.119351709	0.362600722

### Colocalization between MU risk genes and pQTL

**Figure 4:**
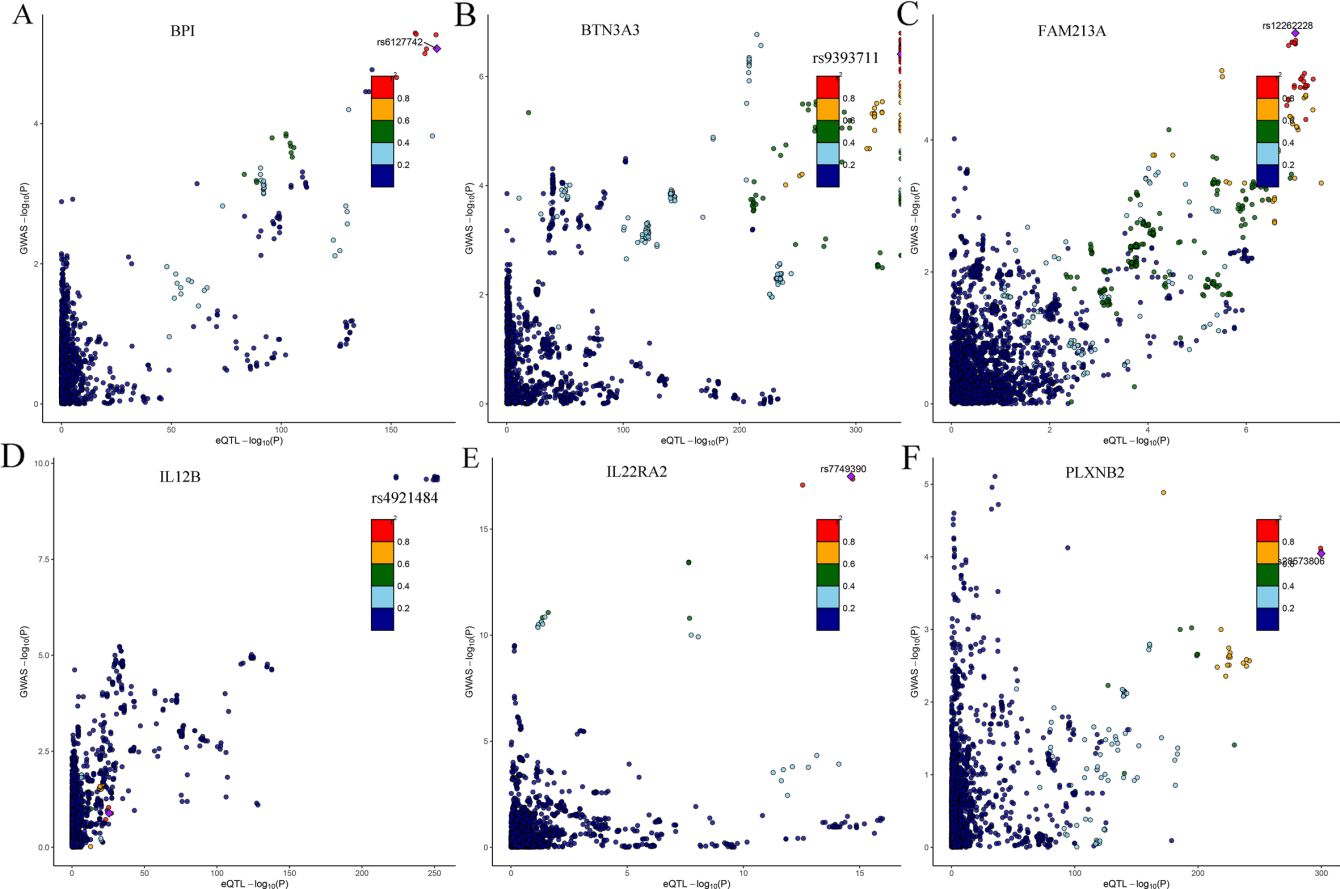
Illustration of the colocalization results. (**A**–**F**) Is for BPI, BTN3A3, FAM213A, IL12B, IL22RA2 and PLXNB2, respectively.

Our study analyzed the posterior probability of a common variant between a pQTL and mouth ulcers for these biomarkers that satisfied the Bonferroni-corrected P-value threshold in previous MR analyses. However, only six genes (BTN3A3, IL12B, BPI, FAM213A, PLXNB2, and IL22RA2) assembled the criterion (PPH4 > 75%) in the analysis of mouth ulcers, indicating a shared single variant with mouth ulcers (Table 2). Through colocalization analysis, six SNP (rs9393711, rs4921484, rs6127742, rs12262228, rs28573806, and rs7749390) were significantly associated with six genes (BTN3A3, IL12B, BPI, FAM213A, PLXNB2, and IL22RA2), respectively (Fig. 4).

**Table 2:** Candidate genes identified by Bayesian colocalization.

FILE	ID	OR	pval	PP.H3.abf	PP.H4.abf	snp
SeqId_17692_2	BTN3A3	1.04	2.90E-06	0.102	0.898	rs9393711
SeqId_13733_5	IL12B	1.09	3.01E-06	0.011	0.989	rs4921484
SeqId_4126_22	BPI	0.96	8.11E-06	0.009	0.983	rs6127742
SeqId_13423_94	FAM213A	1.3	0.000453	0.063	0.915	rs12262228
SeqId_9216_100	PLXNB2	1.04	0.00237	0.068	0.826	rs28573806
SeqId_5087_5	IL22RA2	0.82	0.093313	0.066	0.934	rs7749390

## Discussion

Based on MU GWAS data and blood-derived proteome data, a combination of PWAS, MR, and Bayesian colocalization analysis was used to screen crucial genes for mouth ulcers. PWAS analysis revealed that 35 variations in the gene expression were associated with mouth ulcers. In the MR analysis, 30 variations in the gene expression have a association with mouth ulcers. Ultimately, we identified 6 potential risk genes (BTN3A3, IL12B, BPI, FAM213A, PLXNB2, and IL22RA2) of mouth ulcers with altered protein abundances in the blood. Research into these genes may provide mechanistic and therapeutic targets.

Human genetics research aims to identify therapeutic targets for diseases, which is especially essential for mouth ulcer research. Of these identified genes, IL12B was associated with the risk of recurrent Oral ulcers^20^ and peptic ulcer disease^21^. Meanwhile, IL12B was closed to inflammation development^22,23^. It has been reported that overexpression of BPI inhibits Treg differentiation and intrigues exosome-mediated inflammatory responses in systemic lupus erythematosus^24^. Oh et al., demonstrated that FAM213A is associated with the prognostic significance of tumor development through regulation of oxidative stress, such as myelopoiesis^25^ and oral carcinoma^26^. Zhang et al.^27^ reported that the synergistic effect of CD100 and PlxnB2 promotes the inflammatory response of keratinocytes through the activation of NF-κB and NLRP3 inflammasomes and is involved in the pathogenesis of psoriasis. Several studies demonstrated that IL22RA2 was involved in the inflammation process^28–30^. The expression of several butyrophilin (BTN) and butyrophilin-like (BTNL) molecules was significantly altered by inflammation, including BTN1A1, BTN2A2, BTN3A3, and BTNL8^31^, and associated with tumor development^32–34^.

Several advantages can be drawn from our study. First, Our PWAS for mouth ulcers included the largest and most comprehensive human proteome and GWAS data. Meanwhile, by integrating multidimensional QTL data, we were able to gain a comprehensive understanding of the complex biology of MU in blood. Second, by using Bayesian colocalization, two correlated signals with common causal variants can be estimated at specific sites, and the causative proteins of oral ulcers (BTN3A3, IL12B, BPI, FAM213A, PLXNB2, and IL22RA2) were validated. Finally, this study analyzed protein levels associated with oral ulcers using PWAS. Blood protein screening for mouth ulcers may help provide greater insight into those at high risk for MU recurrence.

There are also some limitations in our study. First, pQTL mapping does not resolve all GWAS signatures. It is difficult to explain how genes are involved in the biological development of oral ulcers at a single level, such as the protein level. It is necessary to conduct more epigenetic studies, such as mQTL analysis, single-cell sequencing, and whole-genome sequencing, to design tailored treatments and fully understand mouth ulcer molecular mechanisms. Second, A larger MU GWAS dataset will be necessary for the validation of our analysis, since we only analyzed one mouth ulcer dataset. Third, it is not sufficient to elucidate the numerous MU GWAS-recognized motifs at the protein and transcriptional levels. To gain a deeper understanding of disease progression, methylation data can be integrated into the analysis. Fourth, considering other races should be taken into consideration when extending our findings. Additionally, this research examines predicted protein levels using an in-silico investigation. To enhance the validation of the results, it would be more desirable to have an independent sample with measured proteomics instead of predicted. Functional genomics and biological experiments must be conducted to elucidate and understand the molecular mechanisms underlying mouth ulcers. Fifth, functional studies and/or genetic evidence suggest that although the identified genetic variations are directly involved in the pathogenesis of mouth ulcers, the underlying mechanisms of the disease are multifactorial, and need to be taken into consideration.

In conclusion, we found strong evidence supporting six novel blood proteins (BTN3A3, IL12B, BPI, FAM213A, PLXNB2, and IL22RA2) associated with mouth ulcers. In our study, we provided suggestions for future biological and therapeutic studies to verify their potential roles in MU.

## Method

The present MR analysis was based on summary data from previous studies^35,36^ that had gained written informed consent and ethics approval. No ethical permit is required for the secondary analysis of summary data.

### Mouth ulcers of GWAS data

A GWAS summary for mouth ulcers was collected from the UK Biobank (UKB) of European ancestry for the present study^7^. By completing questionnaires, and interviews, completing physical measurements, and donating biological samples, participants provided information pertinent to health outcomes in adulthood and later life (data showcase available at http://www.ukbiobank.ac.uk)^37^. The UKB was used for GWAS on mouth ulcers, in which all participants completed a baseline questionnaire regarding oral health. The term "Mouth ulcers (yes/no)" was defined as having had mouth ulcers within the past year.

### Human blood proteomic data

The serum proteomic data was obtained from a large population-based study (Atherosclerosis Risk in Communities (ARIC) study; N ~ 9000)^36^. In 1987 and 1989, 15,792 participants were recruited from four communities in the U.S. for the ARIC study: Forsyth County, North Carolina; suburban Minneapolis, Minnesota; Washington County, Maryland, and Jackson, Mississippi. Blood samples for proteomic measurements were acquired during the third visit (v3) in 1993–1995. After excluding participants without genotype data, the current study retained 9084 participants with plasma protein data. The modified aptamer (‘SOMAmer Reagent’, hereafter referred to as SOMAmers) is a proteomics analysis platform for the determination of serum levels of 4,657 human serum proteins.

### Statistical analysis

#### Proteome‑wide association studies (PWAS)

Using FUSION, PWAS was performed^38^. FUSION was used to estimate the effect of SNPs on protein abundance for proteins with significant heritability (heritability P < 0.01). Several predictive models were used in the analysis, including top1, blup, lasso, enet, and bslmm^38^. A selection of protein weights originated from the comprehensive predictive models. We then integrate the genetic effect of oral ulcers (mouth ulcer GWAS z-score) with protein weights for PWAS of oral ulcers using FUSION. By summing Z-scores and weights of independent SNPs on the locus, a linear sum is calculated. To reduce false positives, Bonferroni-corrected P values were used. Benjamini-Hochberg (BH) method was also used to impute the P value when the false discovery rate was adjusted.

#### Mendelian Randomization (MR) analysis

Through its cis-regulated protein abundance, the PWAS significant genes obtained from the FUSION method were related to mouth ulcers. The most significant genome-wide SNPs (P < 5 × 10^–6^) were targeted and LD clustering was used to determine independent SNPs (R2 > 0.01). Data from QTLs and MU GWAS were harmonized following the same effect alleles. When only one independent QTL is obtained, Wald ratios were employed to estimate the association between the mouth ulcer and genes. In cases where multiple SNPs are available, the ratios of SNP exposures to SNP outcomes were combined using the inverse variance weighting (IVW) method for random-effects meta-analysis. In addition, when the number of genes exceeded three, horizontal pleiotropy was tested using the MR-egger method^39,40^. Bonferroni correction thresholds for the number of genes analyzed were set at P < 0.05/multiple comparisons. Using R version 4.0, the two-sample Mendelian randomization analysis was performed using "TwoSampleMR" version 0.5.5.

#### Bayesian colocalization analysis

To determine whether the same causal signal shared MU risk loci and pQTL, we used the Coloc Bayesian test for colocalization^17^. It was defined that the default COLOC prior should be P1 = 10^–4^, P2 = 10^–4^, and P12 = 10^–5^, where P1 is the probability that a specific variant causes a mouth ulcer, P2 is a measure of the likelihood that a variant in a mouth ulcer correlates with a significant pQTL, and P12 indicates the probability that a specific variant shares common pQTL in a mouth ulcer. Five mutually exclusive hypotheses were tested: H0, no relationship with either GWAS or pQTL; H1, relationship with GWAS and no relationship with pQTL; H2, relationship with pQTL and no relationship with GWAS; H3, relationship with GWAS and pQTL, two independent SNPs; and H4, relationship with GWAS and pQTL, one shared SNP. The posterior probability (PP) method is used to estimate H4 support (denoted as PPH4). Co-localization is defined as strong when PPH4 ≥ 0.75^41^.

## Supplementary Information

Supplementary Table 1.

## Data Availability

The data that support the findings of this study are available from the corresponding author upon reasonable request. Data can be downloaded from the ‘GLIDE’ database (https://data.bris.ac.uk/data/dataset/2j2rqgzedxlq02oqbb4vmycnc2) and blood proteomics data (http://nilanjanchatterjeelab.org/pwas/).
